# Long Noncoding RNA WDFY3-AS2 Represses the Progression of Esophageal Cancer through miR-18a/PTEN Axis

**DOI:** 10.1155/2021/9951010

**Published:** 2021-06-05

**Authors:** Qingling Kong, Guangcai Li, Gang Yin, Kun Li, Dongqing Zhang, Weihao Xu

**Affiliations:** ^1^Hospital Infection Control Office, Rizhao People's Hospital, Rizhao 276800, China; ^2^China-Canada International Health Management Center, Rizhao Hospital of TCM, Rizhao 276800, China; ^3^Department of Clinical Laboratory, Affiliated Qingdao Central Hospital, Qingdao University, Qingdao 266042, China; ^4^Department of Anesthesia, Zhangqiu District People's Hospital, Jinan 250200, China; ^5^Department of Public Health, Zhangqiu District People's Hospital, Jinan 250200, China; ^6^Medical Laboratory Center, Yantai Yuhuangding Hospital, Yantai 264000, China

## Abstract

**Background:**

Understanding the role of lncRNAs in the development of human malignancies is necessary for the targeted therapy of malignant tumors, including esophageal cancer (EC). Nevertheless, the specific role and regulatory mechanism of lncRNA WDFY3-AS2 in EC are still unclear. Here, we examined the functional role and regulatory mechanism of WDFY3-AS2 in EC.

**Materials and Methods:**

RT-qPCR assay was applied to measure the expression of WDFY3-AS2 and miR-18a in EC samples and cells. The luciferase reporter and RIP assays were used to check the relationship between WDFY3-AS2, miR-18a, and PTEN. Counting Clock Kit-8 (CCK-8) assay was carried out to detect cell viability, and transwell assays were used for measuring cell migration and invasion.

**Results:**

Underexpression of WDFY3-AS2 was found in EC specimens and cells, which predicted a poor prognosis of EC patients. Reexpression of WDFY3-AS2 repressed the progression of EC via inhibiting cell proliferation, migration, and invasion. Additionally, WDFY3-AS2 was negatively correlated with miR-18a and positively with PTEN. Furthermore, we discovered that the expression of PTEN decreased by miR-18a mimic was rescued by WDFY3-AS2 overexpression.

**Conclusions:**

WDFY3-AS2 modulates the expressional level of PTEN as a competitive endogenous RNA via sponging miR-18a in EC, which suggests that the WDFY3-AS2/miR-18a/PTEN pathway might be involved in the progression of EC.

## 1. Introduction

Esophageal cancer (EC) is a malignant tumor with rapid progression and poor prognosis and is one of the most fatal diseases worldwide [1]. EC-related risk factors include consumption of foods containing amines nitrite and fungal infection, as well as irritation from hot foods and drinks [2]. Although significant progress has been made in the treatment of EC, such as chemotherapy and surgical resection, the prognosis of EC patients is still poor, and the 5-year survival rate is low, only about 10% [3, 4]. Thus, it is essential to understand the pathogenesis of EC and discover the possible therapeutic targets for EC.

Long noncoding RNA (lncRNA) is a noncoding RNA that cannot encode proteins, and its length exceeds 200 base pairs [5]. Previous reports have discovered that LncRNAs play essential roles in the occurrence and development of EC [6]. In addition, there are enough pieces of evidence that dysregulation of lncRNAs that modulate cancer-related pathways could affect the progression of tumors, including EC [7]. For instance, ZNF750 was involved in ESCC progression and emphasized its significance as a biomarker for metastasis and prognosis [8]. Liang et al. discovered that lnc01980 acted as an oncogenic lncRNA in modulating ESCC proliferation, migration, and invasion [9]. Furthermore, Shen et al. verified that knockdown of AGAP2-AS1 showed suppressive effects on the migration and invasion of EC through miR-195-5p/FOSL1 pathway [10]. LncRNA WDFY3-AS2 was first found to be increased in hepatocellular carcinoma [11]. Moreover, WDFY3-AS2 was decreased in triple-negative breast tumors and served as a potential prognostic factor in TNBC development [12]. Li et al. showed that overexpression of WDFY3-AS2 inhibited tumor growth, cell migration, and invasion [13]. To our knowledge, there are no reports on the role of WDFY3-AS2 in ESCC progression.

Previous literature demonstrated that miR-18a is significantly increased in hepatocellular cancer [14] and gliomas [15]. Also, miR-18a was reported to involve in nasopharyngeal carcinoma by suppressing SMG1 and activating the mTOR pathway [16]. Nair et al. displayed that miR-18a promoted the proliferative and migratory ability of ER-positive breast cancer cells via activating Wnt signaling [17]. In EC, miR-18a was highly expressed in plasma of EC patients [18]. However, there are few reports about the role of miR-18a in EC.

In the present study, we hypothesized that WDFY3-AS2 plays a critical role in regulating EC development with the implication of miR-18a and PTEN. Thus, we investigated the regulatory mechanism of WDFY3-AS2 in the regulation of EC cell proliferation, invasion, migration, and the relationships between WDFY3-AS2, miR-18a, and PTEN. Our findings may provide new insights into the role of lncRNA-miRNA-mRNA in EC.

## 2. Materials and Methods

### 2.1. Patients

Thirty-six EC tissue samples and the corresponding normal tissue samples were collected from EC patients at Rizhao People's Hospital, Rizhao, Shandong, China. None of EC patients received preoperative radiotherapy or chemotherapy before surgery. The tissue samples were stored at −80°C for further use. All patients provided written informed consent and the study was approved by the ethics committee of the Rizhao People's Hospital, Rizhao, Shandong, China (Approval no. 2018020078).

### 2.2. Cell Culture

Immortalized esophageal epithelial cell SHEE and human esophageal cancer cell lines (ECA109, YES2) were purchased from BeNa Culture Collection (Beijing, China). The cells were cultured in RPMI 1640 (Gibco, Invitrogen, Germany) medium containing 10% FBS at 37°C 5% CO_2_. The complete medium was updated regularly every 2-3 days. When the degree of fusion reached 80%, the cells were separated and passaged as usual.

### 2.3. Cell Transfection

The siRNA for WDFY3-AS2 (si-WDFY3-AS2) or PTEN (si-PTEN) was used to knock down WDFY3-AS2 or PTEN. WDFY3-AS2 plasmid was subcloned into the pcDNA3.1 vector to overexpress WDFY3-AS2 (pcDNA3.1-WDFY3-AS2). MiR-18a mimic, miR-18a inhibitor, and corresponding negative controls (NC-mimic and NC-inhibitor) were purchased from GenePharma Company (Shanghai, China). The transfections were performed using by Lipofectamine 2000 reagent (Invitrogen) following the instructions of the manufacturer.

### 2.4. RNA Isolation and Quantitative Real-Time RT-PCR Assay

Total RNA was extracted using TRIzol kit (Invitrogen) following the instructions of the manufacturer. cDNA was generated by Transcriptor First Strand cDNA Synthesis Kit. GoTap®qPCRMaster Mix (Promega) was used to perform RT-PCR. GAPDH and U6 were employed as endogenous controls by using the 2^−ΔΔCt^ method.

### 2.5. Western Blot Analysis

RIPA lysis buffer was carried out to extract total proteins following the instructions of the manufacturer. BCA method was applied to determine the protein concentration. After electrophoresed on 10% SDS-PAGE gel and then transferred to PVDF membranes, the proteins were blocked with 5% skim milk, followed by the primary antibodies at 4°C overnight and secondary antibodies for 1 h at 37°C. Finally, an ECL detection reagent was used to visualize bands.

### 2.6. CCK-8 Assay

ECA109 cells were suspended in a culture medium containing 10% FBS and cultured for 24 h. Then, they were seeded in a 96-well plate with 1 × 10^3^ per well. After incubation for 24, 48, 72, and 96 h, CCK-8 reagents were added and then incubated in a CO_2_ incubator for 2 h. Finally, the optical density at 490 nm was measured.

### 2.7. Transwell Assays

3 × 10^4^ cells/well were added to the upper chamber with a serum‐free medium. The lower chamber was fixed with a fresh medium supplement with 10% FBS. For migration assay, ECA109 cells were incubated at 37°C and 5% CO_2_ for 24 h. The invasion test procedure was the same except that the upper chamber was coated with a matrix. After incubation for 48 h, the migration and invasion cells were stained with 0.1% crystal violet. Finally, a phase-contrast microscope was applied to observe EC cells.

### 2.8. RNA Immunoprecipitation (RIP) Assay

GFP antibody and the Magna RIP™ RNA Binding Protein Immunoprecipitation Kit were carried out to perform RNA immunoprecipitation (RIP) experiments following the manufacturer's instructions. The expressional levels of WDFY3-AS2 and miR-18a were measured by qRT-PCR assay. IgG was used as a negative control.

### 2.9. Luciferase Reporter Assay

The constructed pmirGLO-WDFY3-AS2-WT, pmirGLO-WDFY3-AS2-Mut, pmirGLO-PTEN-WT, or pmirGLO-PTEN-MuT with miR-18a mimic, miR-18a inhibitor, or negative control were cotransfected into ECA109 cells using lipofectamine 2000. After 48 h transfection, the firefly luciferase activity was measured with Dual-Luciferase Reporter Assay System.

### 2.10. Statistical Analysis

The data were shown as mean ± SD and analyzed using SPSS 19.0 or GraphPad Prism 8.0. The statistical differences were assessed by Student's *t*-test or one-way ANOVA. *P* < 0.05 was considered as statistically significant.

## 3. Results

### 3.1. Low Expression of WDFY3-AS2 in EC and Its Clinical Significance

**Figure 1:**
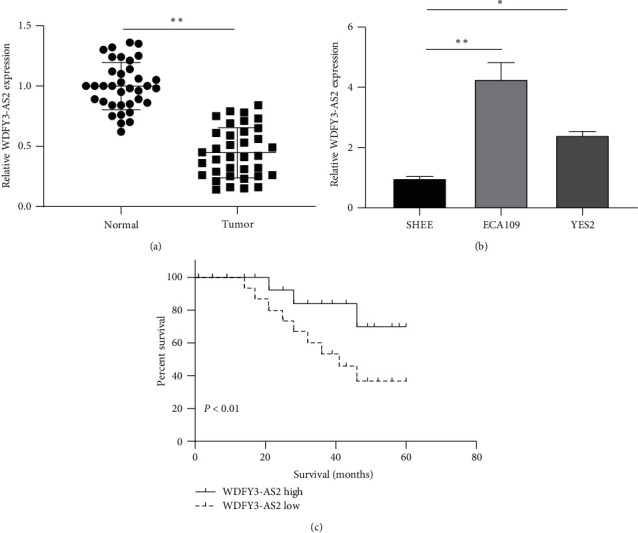
WDFY3-AS2 was decreased in EC. (a) Detection of WDFY3-AS2 expression in EC tissues by RT-PCR. (b) Detection of WDFY3-AS2 expression in EC cell lines by RT-PCR. (c) The overall survival of EC patients with high or low WDFY3-AS2 expression by the Kaplan–Meier analysis. ^*∗*^*P* < 0.05, ^*∗∗*^*P* < 0.01.

We first investigated WDFY3-AS2 expression in EC tissue samples and cells by RT-qPCR. Downregulation of WDFY3-AS2 was found in EC tissue samples compared to corresponding normal tissue samples (Figure 1(a)). Moreover, the expression level of WDFY3-AS2 in EC cells was also higher than that in normal EC cells SHEE (Figure 1(b)). 36 EC patients were divided into a high expression group and a low expression group according to the average of WDFY3-AS2 expression. Results discovered that the higher expression of WDFY3-AS2, the higher the survival time of EC patients (Figure 1(c)). In addition, WDFY3-AS2 is closely associated with tumor grading and TNM stage (Table 1). All results indicate that WDFY3-AS2 is involved in the progress of EC.

### 3.2. Upregulation of WDFY3-AS2 Represses Proliferation, Migration, and Invasion of EC Cells

**Figure 2:**
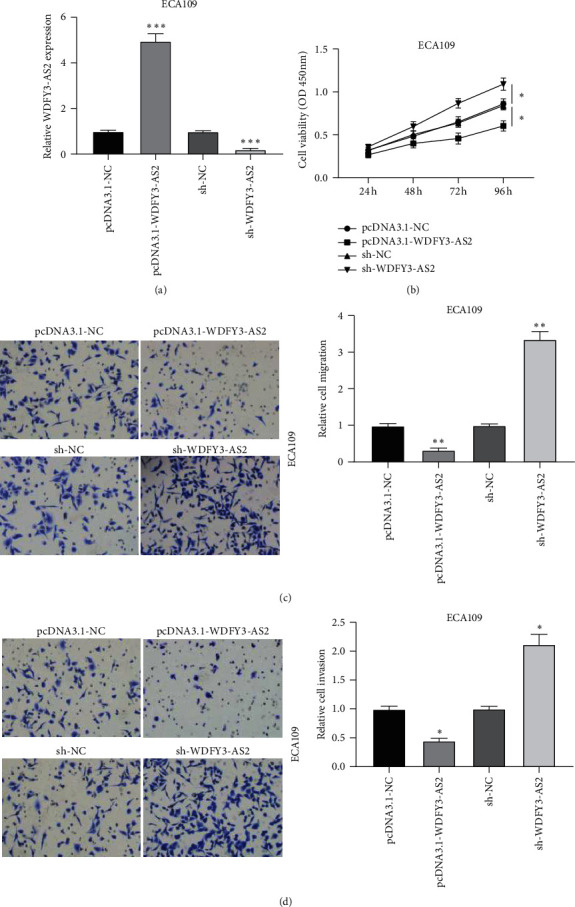
WDFY3-AS2 inhibited EC development. (a) The transfection efficiency of WDFY3-AS2 in ECA109 cells after knockdown and overexpression of WDFY3-AS2. (b) CCK-8 assay revealed WDFY3-AS2 effect on ECA109 cells proliferation. (c) Transwell migration assay revealed WDFY3-AS2 effect on ECA109 cells migration. (d) Transwell invasion assay revealed the cell invasion of ECA109 cells after overexpression or knockdown of WDFY3-AS2. ^*∗*^*P* < 0.05, ^*∗∗*^*P* < 0.01, and ^*∗∗∗*^*P* < 0.001.

Next, we investigated the functional role of WDFY3-AS2 in EC cells. pcDNA3.1-WDFY3-AS2 and sh-WDFY3-AS2 vectors were adopted to overexpress and knockdown WDFY3-AS2 in ECA109 cells. As shown in Figure 2(a), WDFY3-AS2 expression was remarkably increased or decreased in ECA109 cells after overexpression or knockdown of WDFY3-AS2. Then, we detected the viability of EC cells by CCK-8 assay. The ECA109 cells transfected with pcDAN3.1-WDFY3-AS2 displayed significantly decreased cell viability compared to ECA109 cells transfected with pcDNA3.1-NC, while increased cell viability in ECA109 cells transfected with sh-WDFY3-AS2 (Figure 2(b)). Transwell migration and invasion assays displayed that overexpression of WDFY3-AS2 inhibited the migration and invasion of ECA109 cells, whereas knockdown of WDFY3-AS2 exerted the opposite effects (Figures 2(c) and 2(d)).

### 3.3. WDFY3-AS2 Acted as a Sponge for miR-18a in EC

**Figure 3:**
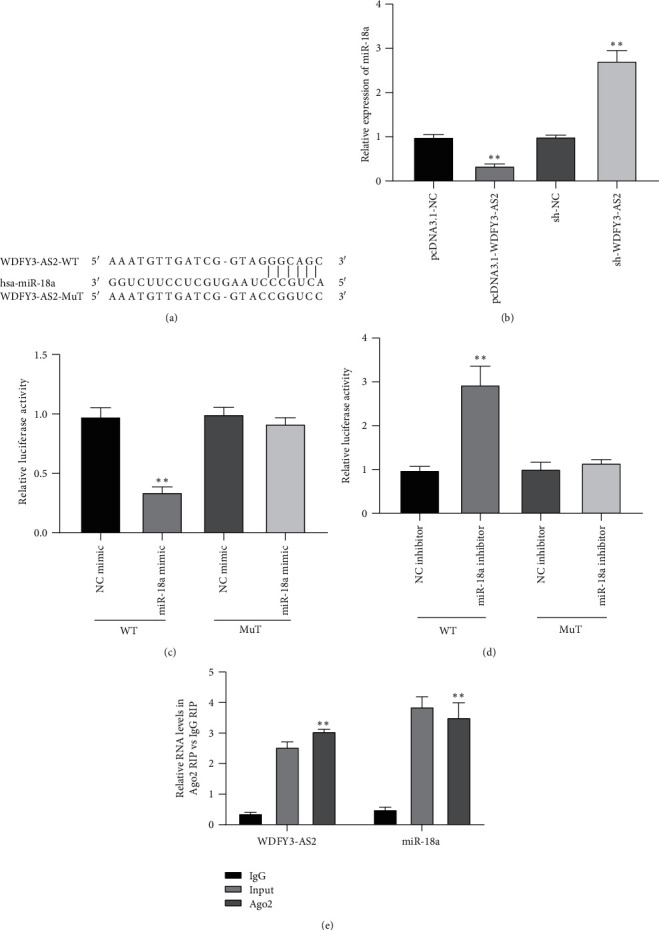
WDFY3-AS2 sponges for miR-18a. (a) The speculated binding sites of miR-18a for WDFY3-AS2. (b) The expressional level of miR-18a affected by WDFY3-AS2 alterations. (c) The luciferase activities of WDFY3-AS2 in response to miR-18a mimic or (d) miR-18a inhibitor. (e) The interplay between WDFY3-AS2 and miR-18a verified by RIP assay. ^*∗*^*P* < 0.05, ^*∗∗*^*P* < 0.01.

To explore the molecular mechanism of WDFY3-AS2 in EC, bioinformatic analysis was first carried out to predict the possible binding site of WDFY3-AS2. miR-18a was obtained as the candidate miRNA, and the predicted binding site of miR-18a in WDFY3-AS2 is shown in Figure 3(a). Then, miR-18a was chosen for the subsequent investigations. Upregulation of WDFY3-AS2 decreased miR-18a expression and downregulation of WDFY3-AS2 increased the expression of miR-18a (Figure 3(b)). Afterwards, luciferase reporter assay and RIP assay were carried to verify the direct binding ability of miR-18a and WDFY3-AS2. The luciferase assay results displayed that the luciferase activity was inhibited or fortified by transfected with WDFY3-AS2-WT and miR-18a mimic or inhibitor (Figure 3(c)). However, there has no response to the alterations of miR-18a in WDFY3-AS2-Mut (Figure 3(d)). RIP assay showed that the expressions of WDFY3-AS2 and miR-18a were abundant in Ago2 groups versus IgG groups (Figure 3(e)). The results demonstrated that WDFY3-AS2 could directly bind to miR-18a in EC.

### 3.4. Overexpression of miR-18a Enhanced Proliferation, Migration, and Invasion of EC Cells

**Figure 4:**
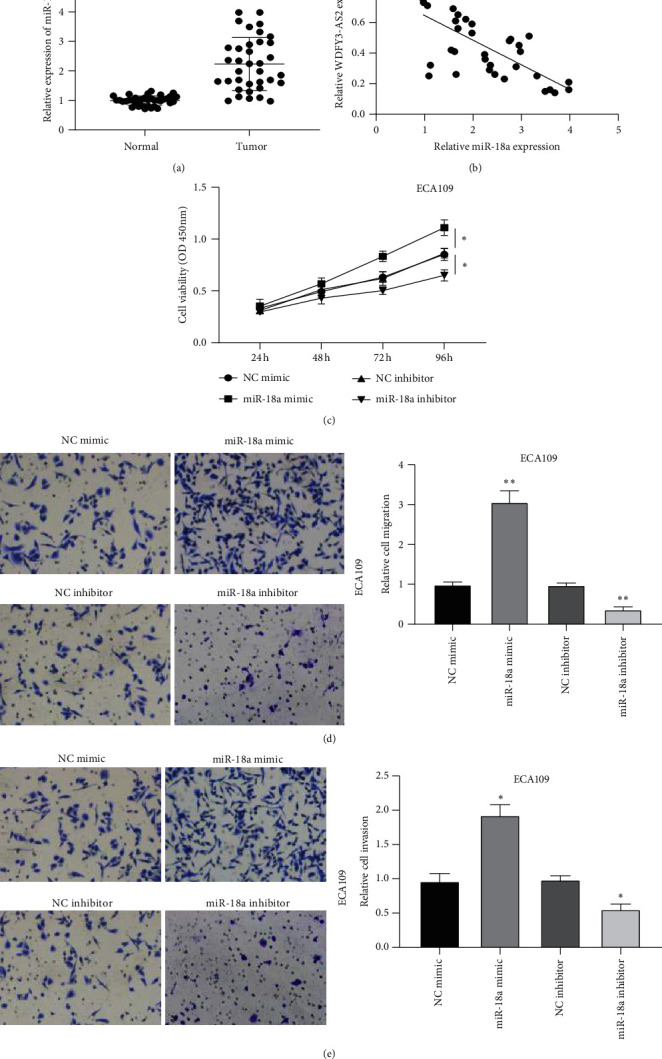
miR-18a enhanced EC development. (a) The expression level of miR-18a in EC tissues. (b) The negative relationship between miR-18a and WDFY3-AS2. (c) CCK-8 assay detected miR-18a effect on ECA109 cells viability. (d) Transwell migration assay revealed miR-18a effect on ECA109 cells migration. (e) Transwell invasion assay detected the cell invasion of ECA109 cells after overexpression or knockdown of miR-18a. ^*∗*^*P* < 0.05, ^*∗∗*^*P* < 0.01.

Next, we detected the expressional level of miR-18a in EC specimens and results found that miR-18a increased significantly in EC samples compared to the normal samples (Figure 4(a)). Considering the negative relationship between WDFY3-AS2 and miR-18a in EC tissues (Figure 4(b)), we investigated the functional role of miR-18a in EC proliferation, migration, and invasion. As shown in Figure 4(c), ECA109 cells transfected with miR-18a mimic displayed significantly increased cell viability compared to ECA109 cells transfected with NC-mimic, while decreased cell viability in ECA109 cells transfected with miR-18a inhibitor. Transwell migration and invasion assays displayed that overexpression of WDFY3-AS2 inhibited the migration and invasion of ECA109 cells, whereas knockdown of WDFY3-AS2 exerted the opposite effects (Figures 4(d) and 4(e)).

### 3.5. PTEN Served as the Target of miR-18a

**Figure 5:**
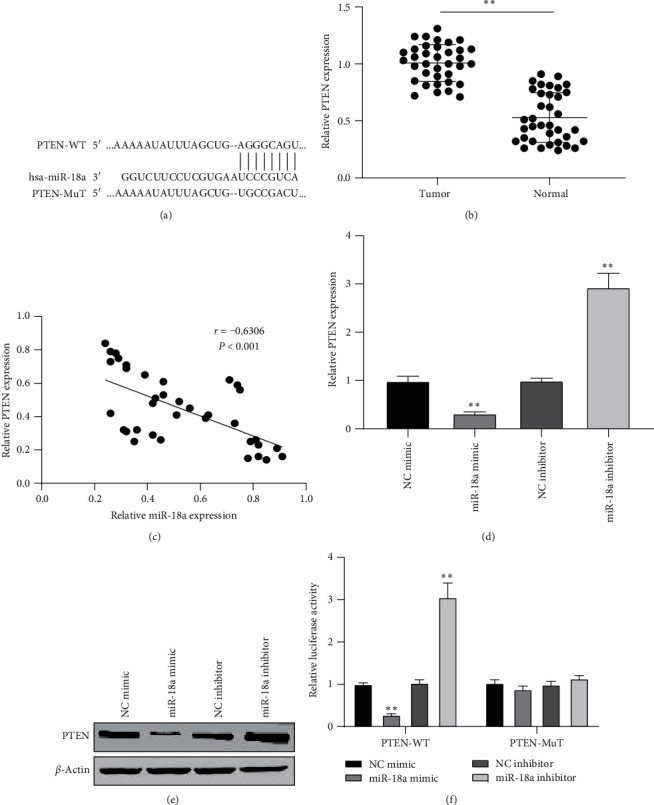
PTEN acted as the target of miR-18a. (a) The prediction binding sites of miR-18a and PTEN. (b) Detection of the expression of PTEN in EC tissues. (c) Pearson's analysis of the relationship between PTEN and miR-18a. (d) The relative mRNA and (e) protein expression affected by miR-18a mimic or inhibitor. (f) The luciferase activity detected in ECA109 cells after treated with miR-18a mimic or inhibitor and PTEN-WT or PTEN-MuT.

TargetScan database was used to discover the target gene of miR-18a, and PTEN was screened as the candidate gene (Figure 5(a)). PTEN was reported to be highly expressed in diverse cancers as an oncogene [19, 20]. We then tested PTEN expression in EC tissue specimens. As shown in Figure 5(b), PTEN was significantly increased in EC tissues. Moreover, we found that miR-18a was negatively associated with PTEN in EC tissues (Figure 5(c)). Overexpression of miR-18a decreased the levels of PTEN in ECA109 cells, while knockdown of miR-18a increased PTEN expression (Figures 5(d) and 5(e)). The luciferase assay results showed that the luciferase activity was significantly inhibited or fortified by being transfected with miR-18a mimic or inhibitor in the PTEN-WT group (Figure 5(f)). These data suggested that PTEN was a direct target of miR-18a.

### 3.6. WDFY3-AS2 Attenuated the Development of EC via Modulating miR-18a/PTEN Axis

**Figure 6:**
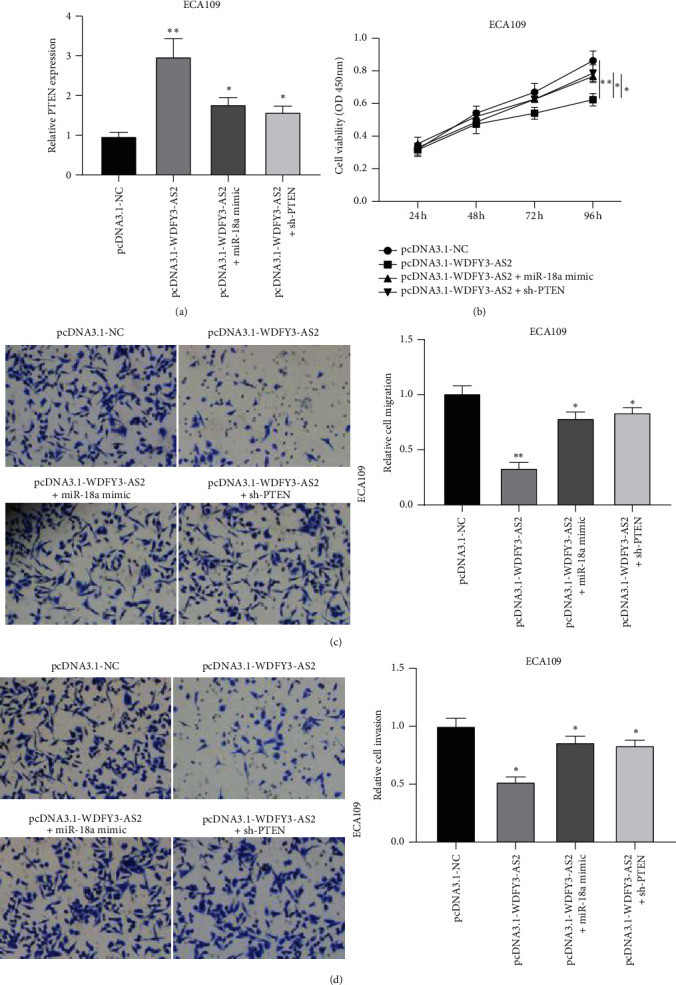
WDFY3-AS2/miR-18a/PTEN axis attenuated EC development. ECA109 cells were transfected with pcDAN3.1-WDFY3-AS2 alone or cotransfected with pcDAN3.1-WDFY3-AS2 and miR-18a mimic or sh-PTEN (a) RT-PCR analysis of PTEN expression. (b) CCK-8 assay to detect ECA109 cells proliferation. (c) Transwell migration assay was performed to investigate ECA109 cells migration. (d) Transwell invasion assay was performed to measure the cell invasion of ECA109 cells. ^*∗*^*P* < 0.05, ^*∗∗*^*P* < 0.01.

Next, rescue assays were conducted to verify the role of WDFY3-AS2/miR-18a/PTEN in EC development. CCK-8 assay discovered that the decreased cell viability caused by overexpression of WDFY3-AS2 was attenuated by upregulation of miR-18a or downregulation of PTEN (Figure 6(b)). Transwell migration and invasion assays demonstrated that EC invasion and migration were repressed by increasing WDFY3-AS2 and restored by upregulation of miR-18a or depletion of PTEN (Figures 6(c) and 6(d)). The results stated that WDFY3-AS2 might inhibit EC progression by regulating PTEN via miR-18a.

## 4. Discussion

Esophageal cancer is a malignant tumor occurring in the esophageal mucosa and is a common gastrointestinal tumor in China [21]. Lack of potential biomarkers and unnoticed early symptoms of potential EC patients often lead to delayed diagnosis [22]. LncRNA has been reported to be involved in the progression and prognosis of various human tumors. In recent years, although lncRNAs have been studied to assist in the clinical diagnosis of cancer as a potential molecular biomarker, the effect is not evident in EC. Previous studies have found that WDFY3-AS2 was underexpressed in EC patients [23], which was consistent with our findings that WDFY3-AS2 decreased in EC tissues and cells. Moreover, we revealed that overexpression of WDFY3-AS2 inhibited cell proliferation, invasion, and migration, whereas inhibited WDFY3-AS2 showed the opposite effect, suggesting the inhibitory role of WDFY3-AS2 in EC progression.

Previous literature has highlighted the critical role of lncRNAs and miRNAs in various biological processes of cancers, including EC [24, 25]. In the current study, we demonstrated that miR-18a acted as a target of WDFY3-AS2. Moreover, WDFY3-AS2 increased PTEN expression by inhibiting miR-18a. These findings were consistent with the previous study that WDFY3‐AS2 binds to miR‐18a and WDFY3‐AS2 negatively regulated miR-18a expression in ovarian cancer cells. MiR-18a was upregulated in various cancers, including EC [26]. However, its functional role in EC has not been clarified. In this study, we discovered that miR-18a was highly expressed in EC tissues and EC cells. MiR-18a promoted EC cells proliferation, invasion, and migration and WDFY3-AS2 attenuated the development of EC via miR-18a/PTEN axis.

The obtained results showed that PTEN was involved in EC progression. PTEN has been reported to have an oncogenic role in a variety of tumors. For instance, Xiang et al. discovered that PTEN repressed cell proliferation and inhibited apoptosis in lung cancer [27]. Besides, PTEN served as a promoter in gliomagenesis by facilitating cell proliferation, tumor growth, and inhibiting apoptosis [28]. Our study confirmed that the reexpression of WDFY3-AS2 suppressed EC cell proliferation, invasion, and migration by increasing PTEN expression via miR-18a. However, our research is in the preclinical stage; the mechanism of action involved is unclear. Therefore, further evaluation of other relevant biomarkers is recommended.

In conclusion, our study suggests that WDFY3-AS2 was underexpressed in EC samples and cells. Low expression of WDFY3-AS2 was associated with poor prognosis of EC patients. Additionally, the reexpression of WDFY3-AS2 suppressed EC progression by miR-18a/PTEN axis. WDFY3-AS2 may be a novel therapeutic and prognostic target for EC.

## Figures and Tables

**Table 1:** Relationship between WDFY3-AS2 expression and clinical characteristics of EC patients.

Item	Cases (*n* = 36)	WDFY3-AS2		*P* value
		Low (*n* = 17)	High (*n* = 19)	
Age (years)				0.709
<60	16	7	9	
≥60	20	10	10	

Gender				0.194
Female	21	8	13	
Male	15	9	6	

Tumor location				0.342
Upper/middle	22	9	13	
Lower	14	8	6	

Tumor size				0.985
<4 cm	17	8	9	
≥4 cm	19	9	10	

Tumor grading				0.018 ^*∗*^
G1	12	9	3	
G2/3	24	8	16	

TNM stage				0.007 ^*∗*^
I-II	13	10	3	
III-IV	23	7	16	

## Data Availability

The datasets used and/or analyzed during the current study are available from the corresponding author on reasonable request.
